# From across the Seas

**Published:** 1916-08-05

**Authors:** 


### FROM ACROSS THE SEAS.

### The Whirlwind Campaign.

Dr. J. A. Hornsby, in an article on the finances of the Small Community Hospital appearing in the *Modern Hospital*, St. Louis, Mo., U.S.A., has, not for the first time, something to say about "whirlwind campaigns" for raising money for institutions. Dr. Hornsby is rather suspicious of the campaigner, not on any question of honesty, but rather because the amount of expense to which an institution is put on account of the campaign is too often an unknown quantity. As to the efficacy of this method of raising funds, he argues that it "is an open question whether the large percentage that these whirlwind money-raisers charge for their work will be an offset to the additional money that they would be able to raise. And yet these campaigners are obliged to charge large percentages for their services; they are obliged to pay a good many expenses, and they indulge in extravagances that the members of the community would not think of, such as the hiring of great numbers of automobiles, the giving of many lunches to many people, and all sorts of campaign dodges to create enthusiasm."

But for the extreme reticence of the British Committee on War Charities it is probable that we might have had before us some particulars of whirlwind campaigns in this country. We have felt and suspected their presence in our midst, but under the existing state of the law their traces and effects have been carefully obscured.

### The Best Profession for a Woman.

In a lecture by Dr. C. T. Anderson on "The Training of Our Girls," published in the *South African Medical Record*, Cape Town, the following anecdote is given apropos of the necessity of suitably instructing girls of the age of adolescence in the physiology of reproduction:—

One experienced teacher told me that at this age most girls fought shy of their mothers and confided in any other sympathetic person, and so she invariably instructed her girls herself to ensure a clean setting, and to implant a real reverence for motherhood. She gave me the following interesting incident: Some girls who had been asked what they meant to be when they grew up gave various answers. One would be a teacher, another a doctor, a third a lawyer, and so on. The teacher said, "You have left out the very best." After further queries, a missionary, a nurse. One girl's face lit up, as she said, "Why, Miss ——— means a mother!" A few days later the mother of one of the girls told her how her daughter had stared at her for a long time that evening, and then said, "I never knew how wonderful you were!"

